# A Retrospective Cohort Study on Human Cystic Echinococcosis in Khyber Pakhtunkhwa Province (Pakistan) Based on 16 Years of Hospital Discharge Records

**DOI:** 10.3390/pathogens11020194

**Published:** 2022-02-01

**Authors:** Huma Khan, Adriano Casulli, Majid Fasihi Harandi, Muhammad Sohail Afzal, Muhammad Arif Nadeem Saqib, Haroon Ahmed

**Affiliations:** 1 Department of Biosciences, COMSATS University Islamabad (CUI), Islamabad 4550, Pakistan; hrk267@hotmail.com; 2 WHO Collaborating Centre for the Epidemiology, Detection and Control of Cystic and Alveolar Echinococcosis, Department of Infectious Diseases, Istituto Superiore di Sanità, 00161 Rome, Italy; 3 European Union Reference Laboratory for Parasites, Department of Infectious Diseases, Istituto Superiore di Sanità, 00161 Rome, Italy; 4 Research Center for Hydatid Disease in Iran, Department of Medical Parasitology, Kerman University of Medical Sciences, Kerman 761, Iran; fasihi@kmu.ac.ir; 5 Department of Life Sciences, School of Science, University of Management and Technology (UMT), Lahore 54782, Pakistan; sohail.afzal@umt.edu.pk; 6 Department of Medical Laboratory Technology, National Skills University Islamabad, Islamabad 4550, Pakistan; arif.nadeem@nsu.edu.pk

**Keywords:** human cystic echinococcosis, clinical epidemiology, Khyber Pakhtunkhwa province, Pakistan

## Abstract

Human cystic echinococcosis (CE) is a worldwide-distributed parasitic zoonotic disease, which represents a threat for both human and animals. The current study aimed at estimating the prevalence of human CE in Khyber Pakhtunkhwa (KPK) province of Pakistan. Clinical records from four major hospitals in this region were reviewed for CE human cases during the period of 2006–2021. Out of 251 (0.00071%) CE patients identified during the considered period, 142 (56.6%) were females, and 109 (43.4%) were males. The highest number of CE cases was recorded in the 21–30 (27.9%) age group, followed by 31–40 (23.1%) and 41–50 (16.3%). Most of the CE patients in KPK province were members of the Afghani ethnic group (17.1%); secondarily, they were Pakistani (6.4%), while for 76.5% ethnicity data were not available. The liver (41%) and the lungs (4.8%) were the most infected organs identified among CE patients in KPK province. The present study identified CE as a significant public health problem in KPK province, and the current findings demonstrated a constant endemicity of CE during the last 15 years. Further filed studies on the active search of CE carriers by means of ultrasound population-based surveys are needed to fill knowledge gaps on clinical and molecular epidemiology of human CE in Pakistan.

## 1. Introduction

Cystic (CE) and alveolar (AE) echinococcosis are zoonotic parasitic diseases caused by the larval stages of tapeworms *Echinococcus granulosus sensu lato* (*s.l.*) and *Echinococcus multilocularis*, respectively. These zoonotic diseases are included in the World Health Organization (WHO) portfolio, which is prioritizing their control [1]. According to 2007 estimates, 1.2 million people are affected globally with loss of 3.6 million Disability-Adjusted Life Years (DALYs) due to human infection [2], and over USD 3 billion economic losses are reported annually [3,4]. In Pakistan, a total cost of USD 4,068,666 (95% CI: USD 3,097,684–5,295,702) was estimated for the year 2017, with USD 3,951,853 (95% CI: USD 2,981,400–5,177,610) attributable to direct diagnosis and treatment-related costs and USD 117,137 (95% CI: USD 91,841–146,979) attributable to wage losses during the treatment period [5]. The life cycle of *E. granulosus s.l.* involves two mammal animal hosts. Canids such as dogs act as definitive hosts, harbouring the adult worm stage in the intestine. Ungulates such as livestock act as intermediate hosts, harbouring the asexual metacestode stage in the abdominal cavity and can be infected through fecal oral route with eggs shed with dog feces [6]. Humans are dead-end hosts, which acquire infection through Echinococcus viable eggs ingestion by contaminated food and water, a hand-to-mouth pathway, or contact with dogs [7]. The ingested eggs hatch in the small intestine and release six-hooked oncospheres, which penetrate the intestinal wall and are transported to various organs or tissues through blood, but echinococcal cysts most commonly develop in the liver and the lungs. The preliminary stage of disease is always asymptomatic or paucisymptomatic, unless complications occur, depending on the size, number, localization, and stage of the cyst. In symptomatic individuals, the chance of cyst rupture is high, and spillage of cyst content (infective protoscoleces) may cause secondary infection [8].

*Echinococcus granulosus s.l.* is a complex of cryptic species that can be identified by molecular approaches. *E. granulosus s.l.* is currently divided in the following species: *Echinococcus granulosus sensu stricto* (*s.s.*) (genotypes G1 and G3), *Echinococcus equinus* (G4), *Echinococcus ortleppi* (G5), *Echinococcus canadensis* cluster (G6/7, G8, and G10), and *Echinococcus felidis* (lion strain). The life cycle of the parasites and their pathogenicity, specificity, transmission dynamics, and epidemiology may differ between genotypes/species. *E. granulosus s.s.* (G1, G3) is considered the most important species causing human cystic echinococcosis (CE) globally [9]. CE is worldwide distributed, but the regions with high endemicity of human CE infection are the Mediterranean region, South America, Asia, and the Middle East including Pakistan and Afghanistan. There is paucity of research on CE in endemic regions of Pakistan [10,11,12]. The land use in Pakistan is pastoral, agropastoral, and agricultural, where human populations live in close association with livestock. Animal husbandry plays a significant role in the economic sustainability and gross domestic product (GDP) of the country. In Pakistan, about 47% of the population is involved in the agricultural sector [13]. Livestock contributes 60% in agriculture with a 12.8% growth in livestock and poultry products [13]. Risk factors for CE infection include free-roaming dogs, home slaughtering, and being a pet owner (dogs) [8,14]. Furthermore, CE transmission may be influenced by climatic and environmental factors affecting the eggs’ survival [15]. 

Studies assessing CE epidemiology have confirmed this zoonotic disease as a major public health problem in central Asia [16]. In fact, 270 million people (58% of the population) are at risk of cystic and alveolar echinococcosis in Central Asia, including Kazakhstan, Tajikistan, Kyrgyzstan, Uzbekistan, Afghanistan, Pakistan, Western China, and Iran [17]. Countries included in the Iranian plateau (Pakistan, Turkey, and Iran) are highly endemic regions. All three neighbouring countries (Afghanistan, China, and India) share socioeconomic and cultural relations with Pakistan, but none of these countries have control programmes for echinococcosis because of the lack of information regarding this zoonotic disease [18]. The surgical incidence rate of human CE in Iran has been estimated at 1.27/100,000 population, with an average of 1295 surgical operations reported annually for the years 2000–2009, and the number of asymptomatic human CE cases in this country was estimated at 635,232 [19]. Based on the Ministry of Health’s official report in Turkey, 52,000 patients underwent CE surgery between 1990 and 2005. The annual CE incidence rate in Turkey ranged from 0.8/100,000 to 2/100,000 [20], with a significant raise in the CE incidence rate until 6.4/100,000 in some regions of the country [21]. A more recent cross-sectional ultrasound population-based study estimated around 106,000 individuals who might be infected by CE in Turkey [22]. The human CE prevalence rate in highly endemic China was estimated at 10.57/10,000 with 47,278 cases of both CE and AE that were recorded in 2018 in 370 different endemic states [23]. 

In the past decades, few epidemiological studies on echinococcosis, like for other neglected tropical diseases, have been conducted in Pakistan, with human CE cases reported from the provinces of Punjab [11,12,24], Sindh [25], and Khyber Pakhtunkhwa [26]. In addition, a recent review highlighted 1702 human CE cases in Pakistan reported between 2000 and 2020 [12]. Khyber Pakhtunkhwa (KPK) is one of most populated provinces of Pakistan, and it is bordering with Afghanistan. The burden of human CE, envisaged for the implementation of control programs, is poorly known in KPK, where the majority of people are working in the agriculture sector and in animal husbandry. In the recent years, the majority of the CE cases have been reported in Afghan people migrating to several localities, including Khyber Pakhtunkhwa (KPK) province [14]. Therefore, the current study was designed to assess the demographic and epidemiological characteristics of human CE cases based on hospital records during a 16-year period in KPK province (Pakistan). 

## 2. Results

**Figure 1:**
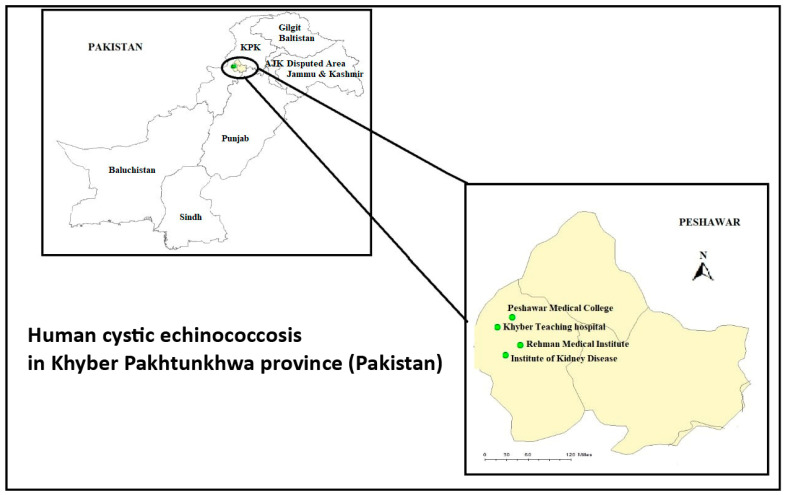
Map of Khyber Pakhtunkhwa province (Pakistan) showing the sites where the study was conducted: (1) Peshawar Medical College, (2) Khyber Teaching Hospital, (3) Rehman Medical Institute, and (4) Institute of Kidney Disease.

In the current study, a total of 251 patients with CE were diagnosed and surgically operated in four major hospitals (Rehman Medical Institute (RMI), Peshawar Medical College (PMC), Institute of Kidney Disease (IKD), and Khyber Teaching Hospital (KTH)) during the 2006–2021 period with 16,56 cases per year (Figure 1). 

**Figure 2:**
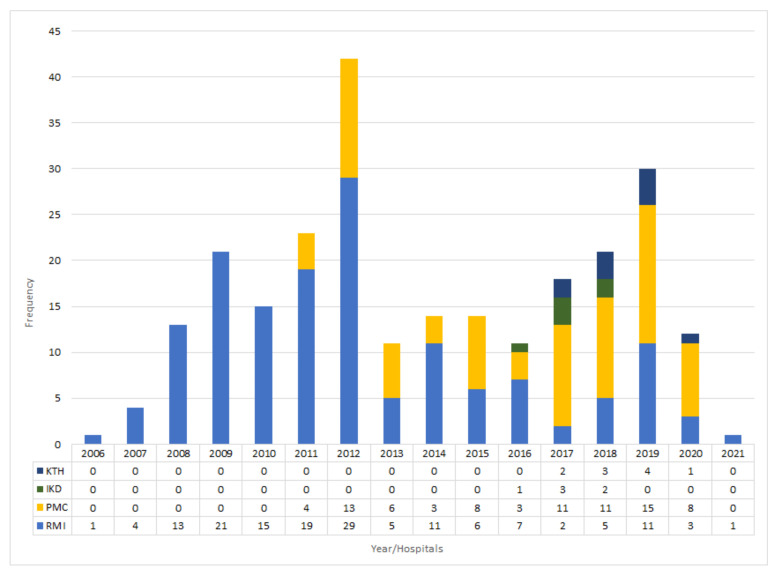
Frequency of cystic echinococcosis in four major hospitals of Khyber Pakhtunkhwa province (Pakistan) during the years of 2006–2021.

The highest number of histopathologically confirmed CE cases were reported in RMI (60.95%; *n* = 153) followed by PMC (32.66%; *n* = 82), while lower number of cases were in KTH (3.98%; *n* = 10) and IKD (2.40%; *n* = 6). The highest annual CE cases were recorded in 2012 (16.73%) followed by 2019 with 11.95%, while the lowest frequencies were recorded in 2006 and 2021 (0.40%) (Figure 2). The current findings were based on clinical records of operated CE patients.

**Figure 3:**
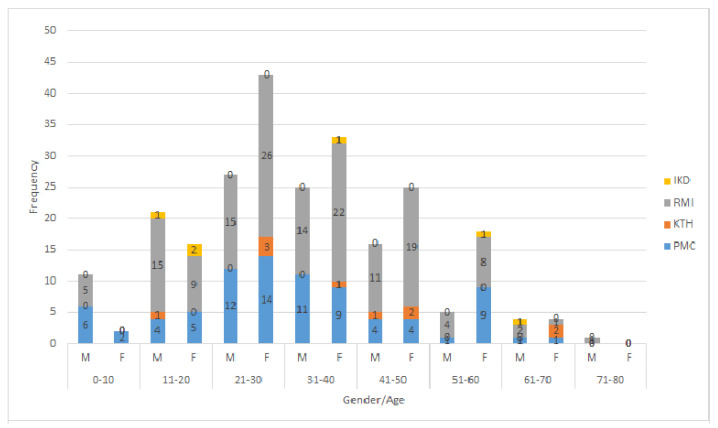
Age- and gender-wise frequency of cystic echinococcosis cases from four major hospitals of Khyber Pakhtunkhwa during the years 2006–2021. M, male; F, female).

Surgery was done after echinococcal cyst identification by diagnostic imaging techniques like, ultrasound (US), computed tomography (CT), or magnetic resonance imaging (MRI). All patients received antihelminthic treatment with albendazole as an adjuvant to surgical procedures. In Pakistan, albendazole is commonly prescribed to all CE patients. The highest number of positive cases (27.9%) were observed in the 21–30 age group followed by the 31–40 (23.1%) and 41–50 (16.3%) age groups, while the lowest cases were recorded in the 71–80 (0.39%) age group. Based on the clinical data, the number of CE cases was observed more in females (56.6%) than males (43.4%). For both genders, most cases were recorded in the 21–30 and 31–40 age groups (Figure 3). 

The cyst localization from four major health complexes of KPK is summarized in Table 1. The CE cases identified in the present study belonged to two ethnic groups. Among the recorded cases, 43 (17.13%) were Afghani, and 16 (6.37%) were Pakistani, while for 192 (76.49%), data were not available. 

The most commonly affected organ was the liver (41%) followed by the lungs (4.78%) and the spleen (3.58%). Seven CE cases were recorded in the abdomen and three cases each in the gall bladder and in the kidney. Various other atypical localizations were identified, such as the acetabulum, brain, breast, cardiac, cervical, hard nodule, hepatogastric, left iliac fossa, neck, omentum, ovary, orbit, parietal, shoulder, and vertebrae. The majority of the patients had single organ involvement (96.67%), while few had multiple organ involvement (3%). Patients with multiple organ involvement included four cases: liver and omentum (0.40%), liver and spleen (0.80%), liver and lungs (1.19%), and liver/spleen/pelvis (0.80%). The diameter of the cysts most commonly measured was ≤4 cm (41.0%), followed by 7–8 cm (17.5%) (Table 2). The reported CE cases and incidence per 100.000 during 2006–2021 are shown in Table 3.

## 3. Discussion

Cystic echinococcosis is an endemic zoonotic disease in many regions of the world, but improved hygiene and by-product management at slaughterhouses (in particular, the disposal of infected organs), better human clinical management, and the implementation of control programmes for CE may decrease *Echinococcus* spp. infections and their sequelae. In Pakistan, the health care system has improved to some extent and brought out many reforms in the last few years but still there are numerous weaknesses like poor administration and a lack of access to trained staff and health policy monitoring. In Pakistan, one of the leading causes of CE in animals is due to socio-cultural practices such as home slaughtering, feeding dogs with the organs of livestock, a lack of deworming of shepherd dogs, and the presence of stray dogs, which are common in Pakistan, favouring the active transmission of the parasite [14]. These conditions enhance the chance of contact, providing ideal conditions for helminthic infections [27]. In addition, there is a lack of information on the burden of CE in Pakistan, since many patients facing financial difficulties do not seek medical attention, and such cases remain undiagnosed and undetected [25]. 

In the current study, the highest rates of infection were reported in the RMI hospital compared to PMC, KTH, and IKD. The highest number of CE cases reported in RMI might be due to the fact that this centre also serves patients from neighbouring Afghanistan. In PMC, a higher number of CE cases was reported in 2019 (*n* = 15) followed by 2012 (*n* = 13), 2018 (*n* = 11), 2017 (*n* = 11), 2015 (*n* = 8), 2013 (*n* = 6), 2011 (*n* = 4), 2016 (*n* = 3), and 2014 (*n* = 3). In RMI, a higher number of cases was reported in the year 2012 (*n* = 29) followed by 2009 (*n* = 21), 2011 (*n* = 19), 2010 (*n* = 15), 2008 (*n* = 13), 2019 (*n* = 11), 2014 (*n* = 11), 2016 (*n* = 7), 2015 (*n* = 6), 2018 (*n* = 5), 2013 (*n* = 5), 2007 (*n* = 4), 2020 (*n* = 3), 2021 (*n* = 1), and 2006 (*n* = 1). The current study showed also a higher frequency of CE cases in the 21–30 year age group followed by the 31–40 and 51–60 groups, respectively. These results are in line with other retrospective studies that showed a higher number of CE infections in the same age groups [25,28,29,30,31]. In the present study, the gender-wise investigation of CE cases revealed females with a high proportion of cases compared to males. These findings are similar to previous studies [26,30,31,32,33], where more females were infected than males, while they are in contrast with [14,34], where more males were infected than females. The present study outcomes toward females may be biased by gender inequalities in Pakistan (social restrictions or a ban to visit hospitals). Moreover, a systematic review and meta-analysis of data has identified "being female" as a potential risk factor, although it is considered as a confounding factor, since some activities conducted by women in rural endemic areas could reflect a higher exposure to the parasite [35]. In fact, questionnaires administered during large cross-sectional study on CE conducted in Bulgaria, Romania, and Turkey did not find any statistically significant difference between gender prevalence [22,36].

For the last forty years, Pakistan has hosted more than 1.4 million Afghan immigrants, who have been forced to flee their homes because of the Soviet war in Afghanistan. Subsequently, the lack of basic facilities like shelter and employment in their home country are serious concerns making their return difficult. Currently, the majority of immigrants in Pakistan are of the Pushtun ethnic group, who live outside refugee camps [13]. The majority of CE cases reported in the present study were Afghan immigrants as documented by a report from hospitals in Khyber Pakhtunkhwa [14,37,38]. Moreover, these immigrants may also bring with them infected animals (e.g., dogs, livestock, and camels) into the country, especially in Khyber Pakhtunkhwa, which might have a major role in the transmission of CE in Pakistan [12,26]. 

Hospital data records from this study showed that in most of the cases, cysts were localized in the liver followed by the lungs. Similar observations were reported in Pakistan and worldwide [11,25]. Moreover, rare anatomical locations of the infection were also found in current study including, the pelvis, spleen, omentum, and ovary, as described by Khan and colleagues [39]. However, we reported atypical cases of CE infection of organs such as the acetabulum, neck, shoulder, and thyroid, which were not previously reported in the area. In the present analysis, the majority of positive CE cases were associated with single organ infection, which was in concordance with previous studies [40,41]. In this retrospective cohort, only few secondary CE infections were recorded. With a primary cyst rupture, released protoscoleces may form new cysts through secondary infection. However, if no cyst clinical history is present, it is difficult to find out whether multiple cysts are primary or secondary infections [25]. Most of the cases reported in the current study had a cyst size range ≤ 4 cm (41.0%) in diameter. Small cysts are usually asymptomatic or paucisymptomatic. When cysts exceed 5 cm, they may complicate and become symptomatic; therefore, they can be more easily diagnosed.

## 4. Materials and Methods

Pakistan is divided into five provinces: Punjab, Sindh, Gilgit Baltistan, Baluchistan, and Khyber Pakhtunkhwa. Khyber Pakhtunkhwa is the third-largest province of Pakistan. Although KPK is the geographically smallest province, it has the third-largest economy and population. The north and the west of KPK province borders with Afghanistan, while the east and the northeast border with Azad Kashmir province (Pakistan), the northern areas with the Kashmir region (Pakistan-administered areas), the southeast with the Punjab state (India), and the southwest with the Baluchistan province of Pakistan. The capital city of KPK is Peshawar, which is the economic centre of the province with a 1257 km^2^ metro area and 1,964,102 million inhabitants [13]. The rural areas of KPK lack medical facilities; therefore, the population of rural areas visit mostly hospitals in the metropolitan area of Peshawar. Therefore, hospital data on human CE were collected from the major city of Khyber Pakhtunkhwa (KPK) province. All selected hospitals in this metropolitan area, such as Rehman Medical College (RMI), Peshawar Medical College (PMC), the Institute of Kidney Disease (IKD), and the Khyber Teaching Hospital (KTH) have also better diagnostic facilities compared to other hospitals and clinics located in rural areas. These hospitals also performed pathological investigation of echinococcal cyst samples received from the surrounding areas. The patient clinical data from 2006 to 2021 included in this study were postoperatively confirmed by histopathology. These four medical centres already had consent from the patients for sample characterization; so, after getting permission from the head of Pathology Departments, we had taken these samples from these hospitals. Age, gender, localization of cyst, size, and cyst number per organ (single or multiple) were recorded from medical reports for each surgically operated CE case. The frequency (%) of human CE cases was calculated by using Microsoft Excel. Histopathologically confirmed cases were collected from the above-cited four major hospitals including RMI, PMC, IKD, and KTH (Figure 1), for which hospital management had already taken consent from each patient at the time of diagnosis/treatment. The samples from the hospitals were collected after getting permission from the head of the pathology department. The current study was approved by ethics committee of COMSATS University Islamabad under no. CUI/Bio/ERB/2021/43.

## 5. Conclusions

The current retrospective study revealed that CE is prevalent in KPK province of Pakistan, indicating CE as a significant public health problem in the study area. Although very limited studies have been conducted on *Echinococcus*/echinococcosis in Pakistan, the prevalence observed in the present study draws attention to CE as a public health concern. Moreover, hospital records are mostly partial since registries are not structured, and they do not report all CE cases managed. The present study shows the emerging trend of echinococcosis among the Afghan immigrants in Pakistan, highlighting the transmission dynamics of the disease. Thus, the present report is a first step towards filling the knowledge gap of CE cases in KPK, Pakistan. Hence, more field-based studies are needed to collect clear epidemiological data on CE prevalence in Pakistan by means of population-based ultrasound surveys (cross-sectional studies).

## Figures and Tables

**Table 1:** Ethnicity and anatomical localization of echinococcal cysts of human origin from Khyber Pakhtunkhwa province, Pakistan.

Ethnicity	Number of Cases	Percentages
Afghani	43	17.13%
Pakistani	16	6.37%
Not available	192	76.49%
**Total**	**251**	
**Body organ**		
Liver	103	41.0%
Liver and Omentum	1	0.40%
Liver/spleen/pelvis	2	0.80%
Lung	12	4.78%
Lung and liver	3	1.19%
Spleen	9	3.58%
Abdomen	7	2.78%
Gall bladder	3	1.19%
Neck	1	0.40%
Omentum	3	1.19%
Ovary	1	0.40%
Right flank	3	1.19%
Right paraspinal cyst	1	0.40%
Shoulder	1	0.40%
Spleen and liver	2	0.80%
Thyroid	2	0.80%
Vertebrae	2	0.80%
Others	23	9.16%
Site not mentioned	72	28.60%
**Total**	**251**	

**Table 2:** Size-wise distribution of single and multiple cysts in human CE cases from Khyber Pakhtunkhwa province, Pakistan.

Number	Size (cm)	Number of Cysts				
		Single	Multiple	Multiple/Single	Not Available	Total
1	≤ 4	10 (9.7%)	39 (37.9%)	-	54 (52.4%)	103 (41.0%)
2	5–6	10 (24.3%)	23 (56.0%)	1 (2.4%)	7 (17.0%)	41(16.3%)
3	7–8	8 (18.1%)	28 (63.6%)	-	8(18.1%)	44 (17.5%)
4	9–10	5 (18.5%)	16 (59.2%)	-	6 (20.7%)	27 (10.7%)
5	11–12	4 (25%)	10 (62.5%)	-	2(12.5%)	16 (6.37%)
6	13–14	1 (16.6%)	4 (66.6%)	-	1 (16.6%)	6 (2.39%)
7	15–16	-	4 (66.6%)	-	2 (33.3%)	6 (2.39%)
8	17–18	1 (50%)	1 (50%)	-	-	2 (0.79%)
9	19–20	1 (33.3%)	2 (66.6%)	-	-	3 (1.19%)
10	21–22	-	1 (50.0%)	-	1 (50.0%)	2 (0.79%)
11	≥23	-	1 (100.0%)	-	-	1 (0.39%)
						**251**

**Table 3:** Number of hospital-recorded cystic echinococcosis cases and their incidence per 100,000 in Khyber Pakhtunkhwa province, Pakistan (2006–2021).

Year	Number of Reported Cases	Total Population *	Incidence Per 100,000
2006	1	18,575,729 ^a^	0.005
2007	4	18,575,729 ^a^	0.021
2008	13	18,575,729 ^a^	0.070
2009	21	18,575,729 ^a^	0.113
2010	15	18,575,729 ^a^	0.080
2011	23	26,000,000 ^a^	0.090
2012	42	26,000,000 ^a^	0.161
2013	11	26,000,000 ^a^	0.042
2014	14	26,000,000 ^a^	0.053
2015	14	26,000,000 ^a^	0.053
2016	11	26,000,000 ^a^	0.042
2017	18	35,525,047 ^b^	0.050
2018	21	35,525,047 ^b^	0.060
2019	30	35,525,047 ^b^	0.084
2020	12	35,525,047 ^b^	0.034
2021	1	35,525,047 ^b^	0.003

* Khyber Pakhtunkhwa population estimated according to demographic surveys at province level ^a^ conducted in 2001 (18.58 million people) and 2011 (26 million people) and by national census ^b^ conducted in 2017 (35.52 million people).

## Data Availability

Not applicable.
